# Factors associated with support for social enforcement of smoke-free policies in Georgia and Armenia

**DOI:** 10.18332/tpc/191510

**Published:** 2024-08-23

**Authors:** Cassidy R. LoParco, Ana Dekanosidze, Arevik Torosyan, Lilit Grigoryan, Varduhi Hayrumyan, Zhanna Sargsyan, Yuxian Cui, Darcey McCready, Regine Haardӧrfer, Nour Alayan, Michelle C. Kegler, Alexander Bazarchyan, Lela Sturua, Marina Topuridze, Carla J. Berg

**Affiliations:** 1 Milken Institute School of Public Health, George Washington University, Washington, USA; 2 Tbilisi State Medical University, Tbilisi, Georgia; 3 Georgia National Center for Disease Control and Public Health, Tbilisi, Georgia; 4 National Institute of Health named after academician S. Avdalbekyan, Yerevan, Armenia; 5 Turpanjian College of Health Sciences, American University of Armenia, Yerevan, Armenia; 6 Rollins School of Public Health, Emory University, Georgia, USA; 7 Petre Shotadze Tbilisi Medical Academy, Tbilisi, Georgia; 8 George Washington Cancer Center, George Washington University, Washington, USA

**Keywords:** tobacco control, smoke-free policy, secondhand smoke exposure, social norms, health communication, risk perceptions, low- and middle-income countries

## Abstract

**INTRODUCTION:**

Armenia’s and Georgia’s high rates of smoking and secondhand smoke and recent implementation of smoke-free laws provide a timely opportunity to examine factors that increase compliance, like social enforcement and support for governmental enforcement.

**METHODS:**

Using 2022 data from 1468 Armenian and Georgian adults (mean age=42.92 years, 48.6% male, 31.6% past-month smoking), multilevel linear regression examined tobacco-related media exposures, social exposures, and perceptions/attitudes in relation to: 1) likelihood of asking someone to extinguish cigarettes where a) prohibited and b) allowed; and 2) support of fines for smoke-free violations (1=not at all to 4=very).

**RESULTS:**

There was low average likelihood of asking someone to extinguish cigarettes where allowed (mean=1.01, SD=1.12) or prohibited (mean=1.57, SD=1.21) and ‘little’ agreement with fines for smoke-free violations (mean=2.13, SD=1.06). Having fewer friends who smoked, greater support for indoor smoke-free laws, and no past-month cigarette use were positively associated with all 3 outcomes. Greater exposure to media and community-based action supporting smoke-free policies, and witnessing more requests to stop smoking where prohibited, were associated with higher likelihood of asking someone to extinguish cigarettes where allowed or prohibited. Less exposure to news stories opposing smoke-free policies and cigarette ads and higher perceived harm of cigarettes were also related to higher likelihood of asking someone to stop smoking where prohibited. Higher perceived harm of cigarettes was also associated with greater agreement with fines for smoke-free violations.

**CONCLUSIONS:**

Comprehensive strategies targeting social norms, media exposure, and risk perceptions are needed to effectively facilitate strategies to enhance smoke-free law enforcement.

## INTRODUCTION

Tobacco use and secondhand smoke exposure (SHSe) are associated with several negative health consequences which disproportionately impact low- and middle-income countries (LMICs)^1^. Armenia and Georgia are two middle-income countries where male smoking rates are among the top 10 highest globally (>50%) but much lower among females (<10%)^2,3^. SHSe is also high in Georgia and Armenia, with >60% of adults reporting past-month SHSe in 2022^4^.

Smoke-free policies are a strong evidence-based tobacco control strategy^5^. Armenia and Georgia recently implemented and started enforcing national comprehensive smoke-free policies in 2022 and 2018, respectively. These policies restrict smoking in public spaces (e.g. parks, indoor workplaces). While there are reductions^6^, SHSe remains high even in places where smoking is prohibited^7,8^. Enforcement of smoke-free policies is crucial, as lack of enforcement undermines their impact and individual perceptions of their effectiveness^9^. Thus, factors related to support and enforcement of these policies among the general population, are crucial and need to be understood. For example, those who do not smoke can request others who are smoking to distance themselves or to stop smoking altogether where it is restricted, providing essential social enforcement for these policies^10,11^. Although research indicates that when asked, individuals will stop smoking in a public area, these requests are not often made^7^. Additionally, fines for smoke-free law violations are effective in increasing compliance^12^. However, factors related to individuals’ intentions to ask others to put out their cigarettes or for support of such fines have been understudied.

Social Cognitive Theory suggests the importance of environmental factors, for example, media exposure and social influence, in shifting one’s perceptions and behaviors^13^. One environmental factor that may be associated with support and enforcement of smoke-free policies is exposure to tobacco-related media^14^. Pro-tobacco media include tobacco advertising (particularly prevalent in LMICs^15,16^) and media opposing tobacco control (e.g. emphasizing negative effects such as cost to hospitality^17,18^), which is associated with positive perceptions and use of tobacco^19^. In contrast, anti-tobacco media (e.g. campaigns promoting smoke-free policies) are associated with increased knowledge, perceived harm, and support for tobacco control, including smoke-free policies^20,21^. Previous research in Armenia and Georgia indicates that exposure to tobacco-related media is associated with risk perceptions, readiness to quit smoking, number of quit attempts, and greater support for smoke-free policies^22,23^.

Support and enforcement of smoke-free policies among the general population may also be impacted by social norms and influences, for example, how prevalent smoking is within one’s social network^24^. Notably, anti-tobacco community mobilization efforts have been effective in increasing adoption and knowledge of smoke-free policies^25^, and role modeling of social enforcement of such laws may impact others’ social enforcement behaviors and support for governmental enforcements efforts^26,27^.

The high rates of tobacco use and SHSe in Armenia and Georgia and the recent implementation of their national smoke-free policies provide a timely opportunity to better understand factors that may increase compliance, such as social enforcement. In this study, we examined the extent to which tobacco-related media exposures, social exposures, and perceptions/attitudes were associated with an individual’s self-reported likelihood of asking someone to put out their cigarette (where prohibited or allowed) and support for fines for smoke-free violations.

## METHODS

### Study overview

Data were from a larger study^25^, as described briefly here. A matched-pairs community randomized controlled trial was used to test the impact of local coalitions on smoke-free policies in Armenia and Georgia. We randomized 28 small- to medium-sized communities (14 per country) to control (assessment only) versus intervention (coalitions, led by local public health centers that were provided financial resources, training, and technical assistance over 3 years). As additional context, some of these communities were also involved in other community-based activities through the Framework Convention on Tobacco Control (FCTC) 2030 initiative, which includes smoke-free policy initiatives^28^; Georgia began participating in 2017 and Armenia in 2020^28^. This study was approved by the Institutional Review Boards of all participating institutions.

### Data collection

In each of the 28 communities, we conducted surveys in 2018 (baseline) and 2022 (follow-up). Current analyses focus on data collected in 2022. The sampling strategies were different in the two countries due to availability of census household data in Armenia (but not in Georgia) and the utility of ‘clusters’ (i.e. geographically defined areas of 150 households) in Georgia (but not in Armenia). In Armenia, addresses in each city were randomly listed and then visited in order until target recruitment was reached. In Georgia, multistage cluster sampling was used (i.e. 5 clusters per city were defined, 15 households per cluster were selected using a random walking method). At each household, potential participants were approached at their homes; the KISH method^29^ was used to identify eligible participants (aged 18–64 years). Those eligible were taken through the consent process and then administered the survey via electronic tablets. The final sample consists of 1468 participants (Armenia=763, Georgia=705).

### Dependent variables

*Likelihood to ask someone to put cigarette out*

Participants were asked: ‘Assuming you wanted someone who was smoking around you to put out their cigarette, how likely would you be to ask them to do so in an area where smoking is [prohibited; allowed]?’ (1=not at all to 4=very likely).

*Agreement with fines for smoke-free violations*

Participants were asked: ‘To what extent do you agree that there should be fines for smokers violating smoking bans?’ (1=not at all to 4=very much).

### Independent variables

Measures for independent variables were drawn or adapted from international surveillance studies^30,31^.

*Tobacco messaging exposure*

Exposure to news stories opposing smoke-free policies (pro-tobacco) was assessed by asking: ‘In the past 6 months, how often have you seen/noticed any news stories talking about the negative aspects of public smoke-free air policies, for example, via the internet, social media (such as Facebook), newspapers, magazines, television, radio, signs, or leaflets?’ (0=never to 3=frequently)^30,31^. To assess cigarette advertising exposure (pro-tobacco), we asked: ‘In the past 6 months, how often have you seen/noticed any advertisements or signs promoting cigarettes [e.g. via the internet, etc.]?’ (0=never to 3=frequently)^30,31^. Exposure to media/messaging supporting smoke-free policies (anti-tobacco) was assessed by asking: ‘In the past 6 months, how often have you seen/noticed: Information about the dangers of smoking cigarettes or information that encourages quitting smoking [e.g. via the internet, etc.]?’. Information about the dangers of being exposed to the smoke of others [e.g. via the internet, etc.]? Any news stories talking about the harms of secondhand smoke or the importance of public smoke-free air policies in your community [e.g. via the internet, etc.]?’ (0=never to 3=frequently)^30,31^. An average of these 3 items was calculated.

*Social exposures*

Exposure to community-based activity supporting smoke-free policies was assessed by asking: ‘In the past 2 years, have you seen any of the following in your community? (Check all that apply; e.g. school-based events; groups of people cleaning parks or stadiums to remove cigarette butts and promote smoke-free policies^25^)?’. A sum score of endorsements to these 6 items was calculated (ranging from 0 to 6). Number of friends who smoke was assessed by asking: ‘How many of your closest friends (who might include relatives and co-workers) smoke cigarettes?’ (0=none to 5=almost all or all)^30,31^. Witnessed someone enforcing smoke-free laws was assessed by asking: ‘In the past 6 months, how often have you witnessed anyone being asked to put out their cigarette in an area where smoking is not allowed?’ (1=never to 4=frequently)^7^.

*Perceptions and attitudes*

Perceived harm of cigarette use was assessed by asking: ‘How harmful to your health do you think the use of regular cigarettes are?’ (1=not at all harmful to 7=extremely harmful)^30,31^. Support of indoor smoke-free laws was assessed by asking: ‘Please rate the extent to which you agree with the following statement: I support the law that prohibits using any tobacco products (including e-cigarettes, IQOS) in indoor workplaces and public places’ (1=strongly disagree to 5=strongly agree)^30,31^.

### Covariates

*Sociodemographics and current cigarette smoking status*

Participants were coded based on their country of residence and asked to report their age, sex, education level, and the number of days in the past 30 days they smoked cigarettes; this was dichotomized into past-month cigarette use (yes vs no).

### Data analysis

We first conducted descriptive analyses to characterize participants. Then, we conducted bivariate analyses (i.e. t-tests, Pearson’s correlation) to examine independent variables and covariates in relation to the dependent variables. Next, we conducted multilevel multivariable linear regression analyses with random intercepts to account for the random effect of community, adjusting for covariates (listed above); betas and 95% confidence intervals were calculated. We ran country-stratified analyses; results showed few differences when compared to overall models, and these differences were likely due to insufficient power. Thus, we presented overall model results. All analyses were conducted in SPSS v26, and alpha was set at 0.05.

## RESULTS

### Participant characteristics

Table 1 provides descriptive and bivariate results characterizing our sample. The average age was 42.90 years (SD=13.55); 51.4% were female, 73.2% were >high school educated, and 31.6% reported past-month smoking. Average exposure was low with regard to news opposing smoke-free policies (mean=0.63, SD=0.88; 0–3=frequently), cigarette advertisements (mean=0.35, SD=0.67; 0–3=frequently), media supporting smoke-free policies (mean=1.21, SD=0.87; 0–3=frequently), and community-based action supporting smoke-free policies (mean=1.08, SD=1.04; sum score ranging 0–6). On average, participants reported that about half of their friends smoked (mean=2.67, SD=1.29; 0–5=almost all or all). Average reports reflected infrequently witnessing requests to stop smoking where it was prohibited (mean=0.76, SD=0.94; 1–4=frequently). Average perceived harm of cigarette use and support of indoor smoke-free laws were high (mean=6.09, SD=1.71; 1–7=extremely harmful; and mean=4.17, SD=1.34; 1–5=strongly agree, respectively). For the outcomes, the average likelihood (1–4=very) to ask someone to put out a cigarette where it was allowed (mean=1.01, SD=1.12) or prohibited (mean=1.57, SD=1.21) was low. The level of agreement with fines for smoke-free violations (1–4=very) was ‘a little’ (mean=2.13, SD=1.06).

**Table 1:** Descriptive statistics and bivariate analyses examining factors associated with support for smoke-free policy enforcement (N=1468)

	*Total (N=1468)*	*Likelihood to ask someone to put cigarette out where allowed*		*Likelihood to ask someone to put cigarette out where prohibited*		*Level of agreement with fines for smoke-free violations*	
	*Mean (SD) or n (%)*	*Mean (SD) or r*	*p*	*Mean (SD) or r*	*p*	*Mean (SD) or r*	*p*
**Sociodemographics**							
**Country**			**<0.001**		**<0.001**		**<0.001**
Armenia	763 (52.0)	1.30 (1.15)		1.71 (1.70)		1.95 (1.12)	
Georgia	705 (48.0)	0.69 (0.99)		1.42 (1.24)		2.33 (0.94)	
**Age** (years)	42.92 (13.55)	0.003	0.910	0.05	0.076	0.03	0.197
**Sex**			**<0.001**		**<0.001**		**<0.001**
Male	713 (48.6)	0.75 (1.02)		1.19 (1.18)		1.74 (1.12)	
Female	755 (51.4)	1.26 (1.16)		1.94 (1.13)		2.50 (0.83)	
**Education level**			**<0.001**		**<0.001**		**0.407**
≤High school	394 (26.8)	0.62 (0.96)		1.18 (1.22)		2.09 (1.08)	
>High school	1074 (73.2)	1.15 (1.14)		1.72 (1.18)		2.14 (1.05)	
**Past-month cigarette use**			**<0.001**		**<0.001**		**<0.001**
No	1004 (68.4)	1.24 (1.15)		1.88 (1.15)		2.45 (0.85)	
Yes	464 (31.6)	0.53 (0.88)		0.90 (1.07)		1.43 (1.13)	
**Pro- and anti-tobacco messaging exposure**							
News stories opposing smoke-free policies^a^	0.63 (0.88)	0.07	**0.010**	0.09	**0.001**	0.04	0.175
Cigarette advertisements^a^	0.35 (0.67)	0.06	**0.029**	0.03	0.191	0.04	0.108
Media supporting smoke-free policies^a^	1.21 (0.87)	0.07	**0.006**	0.25	**<0.001**	0.09	**0.001**
**Social exposure**							
Community-based action supporting smoke-free policies^b^	1.08 (1.04)	0.10	**<0.001**	0.22	**<0.001**	0.12	**<0.001**
Number of friends who smoke cigarettes^c^	2.67 (1.29)	-0.19	**<0.001**	-0.24	**<0.001**	-0.31	**<0.001**
Witnessed a request to someone to stop smoking where not allowed^d^	0.76 (0.94)	0.25	**<0.001**	0.32	**<0.001**	0.07	**0.007**
**Perceptions and attitudes**							
Perceived harm of cigarette use^e^	6.09 (1.71)	0.07	**0.008**	0.18	**<0.001**	0.31	**<0.001**
Support of smoke-free laws including indoor workplaces, public places^f^	4.17 (1.34)	0.10	**<0.001**	0.25	**<0.001**	0.42	**<0.001**
**Support for smoke-free policy enforcement**							
Likelihood to ask someone to put cigarette out where allowed^g^	1.01 (1.12)			0.67	**<0.001**	0.15	**<0.001**
Likelihood to ask someone to put cigarette out is prohibited^g^	1.57 (1.21)	0.67	**<0.001**			0.28	**<0.001**
Level of agreement with fines for smokefree violations^g^	2.13 (1.06)	0.15	**<0.001**	0.28	**<0.001**		

r: correlation coefficient. Bold indicates statistically significant associations.

a Scale of 0=never to 3=frequently.

b Sum score ranging from 0 to 6, with higher numbers indicating more exposure.

c Scale of 0=none to 5=almost all or all.

d Scale of 1=never to 4=frequently.

e Scale of 1=not at all to 7=extremely harmful.

f Scale of 1=strongly disagree to 5=strongly agree.

g Outcomes were measured on a scale ranging from 1=not at all to 4=very. Columns may not total to 100% due to missing values.

### Likelihood to ask someone to put cigarette out where allowed

In bivariate analyses, those reporting higher likelihood to ask someone to put out a cigarette where smoking was allowed reported greater exposure to: news stories opposing smoke-free policies, cigarette advertisements, media supporting smoke-free policies, community-based action supporting smoke-free policies, and requests of someone to stop smoking where prohibited; they also had fewer friends who smoked, higher perceived harm, and greater support for indoor smoke-free laws (all p<0.05). Additionally, they were more likely to be from Armenia, female, >high school educated, and without past-month cigarette use (all p<0.05).

Multivariable linear regression analysis (Table 2) indicated that greater exposure to media (β=0.13; 95% CI: 0.05–0.21) and community-based action supporting smoke-free policies (β=0.09; 95% CI: 0.03–0.14), fewer friends who smoke (β= -0.05; 95% CI: -0.10 – -0.01), witnessing more requests to stop smoking where it was prohibited (β=0.33; 95% CI: 0.27–0.39), greater indoor smoke-free law support (β=0.06; 95% CI: 0.02–0.10), and no past-month cigarette use (β= -0.36; 95% CI: -0.50 – -0.22) were associated with higher likelihood of asking someone to put out their cigarette where allowed. Other related factors included being older (β=0.004; 95% CI: 0.001–0.01) and >high school educated versus ≤high school (β=0.11; 95% CI: 0.0001–0.23).

**Table 2:** Multivariable linear regression models examining factors associated with support for smoke-free policy enforcement

	Likelihood to ask someone to put cigarette out where allowed* (N=1377)			Likelihood to ask someone to put cigarette out where prohibited* (N=1381)			Level of agreement with fines for smoke-free violations* (N=1381)		
	*β*	*95% CI*	*p*	*β*	*95% CI*	*p*	*β*	*95% CI*	*p*
**Intercept**	0.36	-0.11–0.83	0.135	0.15	-0.31–0.61	0.533	1.20	0.84–1.55	**<0.001**
**Sociodemographics**									
Georgia (Ref. Armenia)	-0.48	-0.96–0.003	0.051	-0.13	-0.54–0.29	0.546	0.42	0.20–0.65	**<0.001**
Age (years)	0.004	0.001–0.01	**0.016**	0.01	0.002–0.01	**0.002**	-0.002	-0.01–0.001	0.230
Female (Ref. Male)	0.05	-0.07–0.17	0.442	0.16	0.03–0.30	**0.017**	0.20	0.08–0.32	**0.001**
Education level (Ref. ≤high school)	0.11	0.0001–0.23	**0.049**	0.21	0.09–0.34	**<0.001**	0.07	-0.04–0.18	0.194
Past-month cigarette use (Ref. No)	-0.36	-0.50 – -0.22	**<0.001**	-0.41	-0.56 – -0.26	**<0.001**	-0.51	-0.64 – -0.37	**<0.001**
**Pro- and anti-tobacco messaging exposure**									
News stories opposing smoke-free policies	-0.05	-0.12–0.02	0.154	-0.10	-0.18 – -0.02	**0.013**	0.04	-0.03–0.11	0.259
Cigarette advertisements	-0.003	-0.09–0.08	0.951	-0.18	-0.27 – -0.08	**<0.001**	0.04	-0.05–0.12	0.394
Media supporting smoke-free policies	0.13	0.05–0.21	**<0.001**	0.30	0.22–0.39	**<0.001**	-0.03	-0.10–0.05	0.459
**Social exposure**									
Community-based action supporting smoke-free policies	0.09	0.03–0.14	**0.001**	0.11	0.05–0.16	**<0.001**	0.01	-0.03–0.07	0.603
Number of friends who smoke cigarettes	-0.05	-0.10 – -0.01	**0.016**	-0.06	-0.11 – -0.02	**0.010**	-0.09	-0.13 – -0.04	**<0.001**
Witnessed a request to someone to stop smoking where not allowed	0.33	0.27–0.39	**<0.001**	0.39	0.33– 0.46	**<0.001**	0.04	-0.02–0.10	0.197
**Perceptions and attitudes**									
Perceived harm of cigarette use	0.02	-0.01–0.05	0.156	0.04	0.01–0.08	**0.010**	0.05	0.02– 0.08	**0.003**
Support of smoke-free laws to include indoor workplaces, public places	0.06	0.02–0.10	**0.002**	0.09	0.05–0.13	**<0.001**	0.17	0.13– 0.21	**<0.001**

Bold indicates statistically significant associations.

* Outcomes were measured on a scale ranging from 1=not at all to 4=very.

### Likelihood to ask someone to put cigarette out where prohibited

Bivariate analyses indicated that higher likelihood to ask someone to put out a cigarette where smoking was prohibited correlated with greater exposure to news stories opposing smoke-free policies, media supporting smoke-free policies, community-based action supporting smoke-free policies, and witnessing requests for someone to stop smoking where it was not allowed, as well as having fewer friends who smoke, higher perceived harm, and greater indoor smoke-free law support (all p<0.05). Having a higher likelihood to ask someone to put out a cigarette where smoking was prohibited was also correlated with being from Armenia, female, >high school educated, and without past-month cigarette use (all p<0.05).

In multivariable analysis (Table 2), less exposure to news stories opposing smoke-free policies (β= -0.10; 95% CI: -0.18 – -0.02) and cigarette ads (β= -0.18; 95% CI: -0.27 – -0.08), greater exposure to media (β=0.30; 95% CI: 0.22–0.39) and community-based action supporting smokefree policies (β=0.11; 95% CI: 0.05–0.16), having fewer friends who smoke (β= -0.06; 95% CI: -0.11– -0.02), witnessing more requests to stop smoking (β=0.39; 95% CI: 0.33–0.46), higher perceived harm of cigarettes (β=0.04; 95% CI: 0.01–0.08), greater indoor smoke-free law support (β=0.09; 95% CI: 0.05–0.13), and no past-month cigarette use (β= -0.41; 95% CI: -0.56 – -0.26) were associated with higher likelihood of asking someone to stop smoking where prohibited. Other related factors included being older (β=0.01; 95% CI: 0.002–0.01), female (β=0.16; 95% CI: 0.03–0.30), and >high school educated (β=0.21; 95% CI: 0.09–0.34).

### Level of agreement with fines for smoke-free violations

In bivariate analyses, higher level of agreement with fines for smoke-free violations was associated with greater exposure to media and community-based action supporting smoke-free policies, witnessing more requests to stop smoking, having fewer friends who smoked, higher perceived harm, and greater indoor smoke-free law support (all p<0.05). They were also more likely to be from Georgia, female, and without past-month cigarette use (all p<0.05).

Multivariable analysis (Table 2) indicated that having fewer friends who smoked (β=0.09, 95% CI: -0.13 – -0.04), higher perceived harm of cigarettes (β=0.05; 95% CI: 0.02–0.08), greater indoor smoke-free law support (β=0.17; 95% CI: 0.13–0.21), and no past-month cigarette use (β= -0.51; 95% CI: -0.64 – -0.37) were associated with greater agreement with fines for smoke-free violations. Other related factors included being from Georgia (β=0.42; 95% CI: 0.20–0.65) and female (β=0.20; 95% CI: 0.08–0.32).

## DISCUSSION

In this sample of adults in Armenia and Georgia, self-reported likelihood of asking someone to put out their cigarettes – either in a place where it was prohibited or allowed – was low. This aligns with prior research that, despite people’s willingness to extinguish their cigarettes if asked, they are infrequently asked^7^. This is likely due to a desire to avoid confrontation or potential conflict, or perceptions that it is not a person’s responsibility to enforce smoking regulations. This is problematic, given that social enforcement is a powerful tool to increase policy compliance^10,11,32^. The literature suggests that comprehensive smoke-free policies will eventually become socially acceptable and complied with, people will become more assertive in dealing with public violations, and support for smoke-free policies increases once implemented^10,33,34^. Indeed, those in Georgia (where the smoke-free law was implemented 4 years earlier than Armenia) were more supportive of fines for smoke-free law violations but were not more willing to socially enforce the laws. Thus, additional efforts may be needed to promote and enforce the policies; for example, people’s confidence in socially enforcing smoke-free policies and fostering a culture of compliance could be enhanced by interventions that involve modeling of effective social enforcement interactions/activities (e.g. mini dialogues via media campaigns), as well as more visible, active government enforcement^26,27^. Qualitative research may also be beneficial to assess underlying social-cultural factors that may enhance the development and implementation of such social norms interventions.

Regarding theory-based factors, all three outcomes – likelihood of asking someone to put out their cigarette where it was allowed and where prohibited and support for fines for smoke-free violations – were undermined by having more friends who smoked, being less supportive of indoor smoke-free laws, and personally using cigarettes (as well as being male and less educated), which have been well-documented factors impacting enforcement-related perceptions and behaviors^35^. Thus, focused intervention messages are needed to address how to manage one’s own smoking behaviors and negotiate such behaviors within one’s social network (e.g. restricting locations for smoking), especially as smoke-free laws are implemented and social norms shift.

Importantly, there was considerable overlap between factors associated with individuals’ self-reported likelihood of asking an individual to stop smoking in a place where it was allowed and prohibited, specifically greater exposure to media and community-based action supporting smoke-free policies, and witnessing more requests to stop smoking where it was prohibited. These findings are promising and indicate the utility of civil society, mass media campaigns, and role modeling to change not only perceptions, but also behavior^26,27^.

Interestingly, there were unique factors related to likelihood to request that others stop smoking where smoking was prohibited. Specifically, greater exposure to pro-tobacco news and cigarette ads, as well as perceived harm of cigarettes, were associated with lower likelihood to ask individuals to stop smoking in areas where smoking was prohibited – but not where it was allowed. This might suggest that people’s willingness to socially enforce smoke-free laws and their support for legal enforcement are more malleable relative to people’s general tendency to advocate for themselves, which also points to the importance of comprehensive tobacco control legislation that collectively addresses both smoke-free laws and tobacco advertising in order to effectively shift social norms and attitudes^5,36^.

### Strengths and limitations

Study strengths include comprehensive assessment of relevant factors, spanning across several domains (e.g. industry messaging, social contexts, attitudes). The study sample includes data from two countries that recently implemented nationally comprehensive smoke-free laws, making the data and analyses timely and generalizable. Study limitations include analysis of cross-sectional data and thus inability to determine causality, potential recall bias (e.g. self-report of media exposure), unmeasured confounders, small effect sizes in some cases, and limited generalizability of these data to the populations of these countries, as participants were from small- to medium-sized communities (e.g. excluding those in the largest cities and highly rural areas).

## CONCLUSIONS

Collectively, current findings reflect the importance of comprehensive strategies targeting social norms, media exposure, and individuals’ risk perceptions to effectively facilitate strategies to enhance enforcement of smoke-free laws. Thus, when implementing nationally comprehensive smoke-free policies, it is essential to change the smoking culture and related norms at the community level (e.g. via mass media campaigns, community coalitions) and by encouraging strategies within one’s social networks (e.g. pledging with friends/family to stop smoking, implementing smoke-free rules in personal settings).

## Data Availability

The data supporting this research are available from the authors on reasonable request.
